# 

**Published:** 1947

**Authors:** 


| | Page |
|---|---|
| The Thirty-fifth Long Fox Memorial Lecture: Some Aspects of Aviation Medicine. By K. G. Bergin, M.A., M.D., A.F.R.Æ.S., *A.D.M.S., B.O.A.C.* | 31 |
| Penicillin in Diseases of the Ear, Nose, and Throat. By E. Watson-Williams, *M.C.*, Ch.M. | 42 |
| Cardiac Anomalies Associated with Funnel Chest. By G. E. F. Sutton, *M.C.*, M.D., M.R.C.P. | 45 |
| Reviews of Books | 49 |
| Editorial Notes | 51 |
| Non-Editorial Note | 52 |
| Obituary. Stanton Bryan Adams, M.B., Ch.B. B.Sc., Ph.D. (Brist.) | 54 |
| Meetings of Societies | 55 |
| The Medical Library of the University of Bristol | 55 |
| Publications Received | 57 |
| Local Medical Notes | 58 |

